# A Systemic Evaluation of Cardiac Differentiation from mRNA Reprogrammed Human Induced Pluripotent Stem Cells

**DOI:** 10.1371/journal.pone.0103485

**Published:** 2014-07-28

**Authors:** Ashish Mehta, Vinod Verma, Manasi Nandihalli, Chrishan J. A. Ramachandra, Glen L. Sequiera, Yuliansa Sudibyo, Yingying Chung, William Sun, Winston Shim

**Affiliations:** 1 National Heart Research Institute Singapore, National Heart Centre, Singapore, Singapore; 2 Experimental and Therapeutics Centre, A’STAR, Singapore, Singapore; 3 Cardiovascular and Metabolic Disorders Program, DUKE-NUS, Singapore, Singapore; University of Tampere, Finland

## Abstract

Genetically unmodified cardiomyocytes mandated for cardiac regenerative therapy is conceivable by “foot-print free” reprogramming of somatic cells to induced pluripotent stem cells (iPSC). In this study, we report generation of foot-print free hiPSC through messenger RNA (mRNA) based reprograming. Subsequently, we characterize cardiomyocytes derived from these hiPSC using molecular and electrophysiological methods to characterize their applicability for regenerative medicine. Our results demonstrate that mRNA-iPSCs differentiate ontogenetically into cardiomyocytes with increased expression of early commitment markers of mesoderm, cardiac mesoderm, followed by cardiac specific transcriptional and sarcomeric structural and ion channel genes. Furthermore, these cardiomyocytes stained positively for sarcomeric and ion channel proteins. Based on multi-electrode array (MEA) recordings, these mRNA-hiPSC derived cardiomyocytes responded predictably to various pharmacologically active drugs that target adrenergic, sodium, calcium and potassium channels. The cardiomyocytes responded chronotropically to isoproterenol in a dose dependent manner, inotropic activity of nifidipine decreased spontaneous contractions. Moreover, Sotalol and E-4031 prolonged QT intervals, while TTX reduced sodium influx. Our results for the first time show a systemic evaluation based on molecular, structural and functional properties of cardiomyocytes differentiated from mRNA-iPSC. These results, coupled with feasibility of generating patient-specific iPSCs hold great promise for the development of large-scale generation of clinical grade cardiomyocytes for cardiac regenerative medicine.

## Introduction

The ability to revert mature differentiated cells to pluripotent state by the ectopic expression of key transcriptional factors [1]–[3] has opened up the prospects that patient specific cells could be turned into various cell types for autologous regenerative therapies aimed at repairing defects from injury, illness or aging. While induced pluripotent stem cells have unlimited proliferative potential, utilizing viral vectors to deliver transcriptional factors like retrovirus or lentivirus have significantly hampered their therapeutic applications due to the concerns of insertional mutagenesis, tumourigenesis and continued expression of potentially oncogenic proteins by the integrated transgenes [4], [5]. These concerns are particularly important when considering clinical translation. Currently, most of the patient specific iPSC lines derived have utilized genome integrating methods [6]–[10]. It is therefore desirable to generate iPSC using non-integrating based protocols, whilst being robust and fully compliant to clinical requirements like GMP.

Considerable progress has been made in the development of novel strategies to delivery reprograming transcriptional factors without genetic integration. Multifactor encoding polycistronic cassettes flanked with recombination sites in lentiviral vectors that allows excision of the reprogramming transgenes post reprogramming by transient expression of recombinase [11]–[14]. Transgene insertion and excision can also be performed by a transposon vector with a transposase [15], [16]. Non-integrating vectors based on plasmids and episomal DNA have been efficiently developed to express the transcriptional factors to generate iPSC [17]–[21]. However, episomal vectors have been considered to be superior to the traditional plasmids in terms of their increased expression duration of the reprograming factors in the target cells. Apart from the DNA vectors, repeated transduction of reprograming factor proteins have shown to generated iPSC with low efficiencies from mouse fibroblast [22]. However, this strategy of recombinant proteins may be challenging as bacterial systems may not be ideal for proper post translational modifications. Relatively high efficiency can be achieved using Sendai virus, a RNA based virus [23], [24], eliminating any chance of genomic integration. However, integration free, virus-free and vector-free hiPSCs can be derived by sustained transfection of synthetic modified mRNA with high efficiencies, with zero “foot prints” [25].

While generation of hiPSC using synthetic modified RNA have been documented [25]–[28], there is limited information on their differentiation bias towards various cell types, particularly cardiomyocytes. We have previously shown that iPSC could be generated using episomal based systems, however, their low efficiency and longer time frames may hamper clinical applications [29]. Thus the present study was planned to a) utilize mRNA reprogramming methods to generate iPSCs and characterize their cardiomyogenic potential, b) define developmental steps involved in differentiation and c) to study molecular, structural and functional properties of mRNA-iPSC derived cardiomyocytes (iPSC-CMs).

## Materials and Methods

### Cell culture and reprogramming of fibroblasts

Commercial human fibroblasts (Stemgent, MA, USA) and dermal fibroblasts from a 57 year old, male cardiac patient with no specific genetic pre-disposition were cultured in fibroblast culture medium [30]. Fibroblasts were passaged every 4–5 days with TrypLE (Invitrogen, CA, USA) and seeded in new flasks at a density of 15,000–20,000 cell/cm [31]. Patient fibroblast were obtained from skin biopsy after approval from the Institutional Review Board (IRB) of the host institute.

Reprogramming of human BJ and normal patient fibroblasts were performed using mRNA reprogramming kit (Stemgent, MA, USA) as per the recommended protocol. Briefly, inactivated new-born foreskin fibroblasts (NuFFs, Stemgent, USA) were plated at the density of 0.25 m cells per well in growth media containing High Glucose DMEM with 10% FBS (Hyclone) and Penicillin- streptomycin- Glutamine (Invitrogen CA, USA). Human fibroblast cells (Stemgent, MA, USA) were seeded on inactivated NuFF. On the first day of transfection, medium was switched to pluriton reprogramming medium (Stemgent, MA, USA) and B18R (200 ng/ml). The cells were incubated with B18R for 4 hrs at 37°C and 5% CO_2_. mRNA cocktail prepared has molar stoichiometry of 3∶1∶1∶1∶1∶1 for OSKML (Oct4, Sox2, Klf4, c-myc and Lin28) and nGFP mRNAs. RNA transfection was carried out with RNAi Max reagent according to manufacturer instructions. Target cells were transfected daily with mRNA factors for 17 consecutive days using RNAi Max reagent. On day six of transfection the pluriton medium was switched to NuFF pluriton conditioned medium which was supplemented with bFGF (4 ng/ml, Invitrogen, CA, USA) and Penicillin and Streptomycin for 9–10 days. Colonies were picked and passaged on mitomycin C inactivated MEFs. Primary iPSC colonies were switched from conditioned medium to normal hESC cell medium (DMEM F12, 20% Knock out serum replacer, non-essential amino acids, penicillin streptomycin glutamine and 0.1 mM β-mercaptoethanol). Live-cell staining for TRA-1-60 and/or TRA-1-81 was performed for the identification of bona fide iPSC colonies (Stemgent, MA, USA). Twelve clones were manually picked and passaged and maintained on matrigel in chemically defined mTeRS1 medium as reported previously [29], [32].

### Embryoid body formation and cardiomyocyte differentiation

Pluripotent stem cells colonies were dispersed into small clumps with dispase (1 mg/ml) and placed in low adhesion culture dishes in EB medium [33] along with or without 5 µM of SB203580 (Calbiochem, USA) for 8 days as reported previously [29], [31]. Subsequently, EBs were plated on 0.1% gelatin coated dishes in EB media without SB203580. Beating areas were typically observed around day 11–14 from EB formation. Cells were used for various experiments. However, specifically for MEA recording, beating areas were manually cut after day 21 of differentiation and maintained in culture for about 6–8 weeks before as described previously [32].

### RNA extraction and PCR

For semi-quantitative RT-PCR, 5 ng cDNA template was used for each sample and PCR was performed for 30 cycles 95°C for 15 sec, 60°C for 30 sec and 72°C for 30 sec, with initial deactivation at 95°C for 5 min and final extension at 72°C for 7 min in GeneAmp PCR system 2700 (Applied Biosystems, USA). PCR products were electrophoresed on 1.5% agarose gel with ethidium bromide (Sigma-Aldrich, MO, USA) and bands were visualized and recorded using Geldoc XR (Bio-Rad, USA).

For real-time reverse-transcription polymerase chain reaction (qRT-PCR) analysis, undifferentiated hiPS/hES cells (day 0) and differentiating EBs at different time points were utilized. RNA was isolated with the RNeasy kit (Qiagen GmbH, Hilden, Germany). One µg of total RNA was converted to complementary DNA by Superscript II first-strand synthesis system (Invitrogen, CA, USA). Complementary DNA (cDNA) template (5 ng) was used from each sample and SYBR green real-time PCR studies were performed using Quantifast kit (Qiagen GmbH, Hilden, Germany) and primers (supplementary table 1) as per the kit instructions. Samples were cycled with Rotor-Gene Q (Qiagen GmbH, Hilden, Germany) as follows: 5 minutes at 95°C, followed by 40 cycles of 10 seconds at 95°C and 30 second extension at 60°C. All experiments were performed in triplicates. Relative quantification was calculated according to the ΔΔCt method for quantitative real-time PCR (using an endogenous control gene, GAPDH). For each gene, the expression at a specific day was then normalized by its baseline values.

### Immunostaining and immunohistochemistry

Colonies of iPSC and single cells generated from beating clusters were seeded on matrigel and gelatin coated glass slides, respectively. Both cell types were fixed with 4% paraformaldehyde, permeabilized with 0.1% Triton X-100 (Sigma-Aldrich, MO, USA) and blocked with 5% bovine serum albumin (Sigma-Aldrich, MO, USA). Human iPS colonies were stained for 1 hour with primary antibodies targeting pluripotency markers, Oct-4, Sox2, Nanog, SSEA4, Tra-1-60 and Tra-1-80 (all at 1∶200 dilution; Millipore, MA, USA), whereas cardiomyocytes (CMs) were stained with primary antibodies, Nkx2.5 (1∶200; Santa Cruz Biotechnology, CA, USA), α-actinin (1∶200; Sigma-Aldrich, MO, USA), MLC2a (1∶200; Synaptic System, Germany), titin (1∶200; Sigma-Aldrich, MO, USA), cardiac troponin T (1∶200; USBiologicals, MA, USA), connexin 43 (1∶200; Sigma-Aldrich, MO, USA) and SERCA (1∶200; Sigma-Aldrich, MO, USA). Samples were washed and incubated with respective secondary antibodies (1∶400; Invitrogen, CA, USA) for 1 hour and subsequently counterstained with DAPI. Slides were examined under Zeiss LSM710 NLO multi-photon confocal microscope (Carl Zeiss Inc, USA).

Standard IHC protocol was followed to stain the EBs. In brief, 5 µm sized paraffin embedded tissue sections were de-paraffinized with xylene and endogenous peroxidase activity was quenched with 3% H_2_O_2_ in methanol for 30 minutes in dark. Tissue sections were dehydrated through graded alcohols and subjected to antigen retrieval using 10 mM sodium citrate. Sections were washed with PBST (Phosphate buffered saline-tween20) and then blocked with 5% BSA (Bovine serum albumin) for one hour. Slides were incubated with primary antibodies at (1∶200 dilution) overnight. Slides were washed for 5 minutes in PBST and incubated for 1 hour with secondary antibody tagged to HRP (1∶400; Life Technologies, CA, USA) ratio. After washing, slides were incubated with DAB (3,3′- diaminobenzidine tetrahydrochloride) for color development, mounted and observed under microscope.

### Teratoma formation

All animal experiments were conducted following experimental protocols approved by the SingHealth Institutional Animal Care and Use Committee, in full compliance with Singapore laws and regulations and followed the guidelines by US National Institutes of Health Guide for the Care and Use of Laboratory Animals. Severe combined immunodeficient (SCID) mice, 6-w old, weighing 20–23 g, were obtained from SingHealth Experimental Medicine Centre (SEMC) and were anesthetized with 2% isofluorane initially followed by 1% isofluorane during surgery. Approximately 1×10^6^ hiPS cells, were injected into the kidney capsule as previously described [34]. Mice were euthanized with carbon dioxide asphyxiation at 8 weeks after cell injections and tumors were collected, fixed and processed for H&E staining following conventional protocols.

### Microelectrode Array (MEA) Recordings

To characterize the electrophysiological properties of the hiPS-CMs, a microelectrode array (MEA) recording system (Multichannel Systems, Reutlingen, Germany) was used. Contracting areas were micro-dissected and plated on gelatin coated MEA plates. The clusters were allowed to adjust for 72 hrs before performing any recording. All clusters were monitored for their beating abilities (beats/min) under the microscope during the 72 hrs period. Clusters that maintained relatively uniform beating rates were then subjected to drugs. The MEA system allows simultaneous recordings from 60 titanium nitride– coated gold electrodes (30 µm) at high spatial (200 µm) and temporal (15 kHz) resolutions. To assess the effects of different drugs on the electrophysiological properties, the stock drugs were diluted in medium (2 mL). MEA clip along with the beating clusters was maintained on 37°C throughout the duration of experiments. Care was also taken that all buffers including the medium utilized during all experimentation were pre-warmed to 37°C. The tested drugs include isoproterenol hydrochloride, carbamylcholine, verapamil, Bay K8644, tetrodotoxin, Nifedipine, Sotalol and E-4031 (all from Sigma-Aldrich, MO, USA). All extracellular recordings were performed for 180 seconds at baseline and at 5 minutes after drug application at 37°C. Data was recorded using MC Rack software (Multichannel System, Germany) for all drugs. The recorded electrograms were also used to determine the local field potential (FP) duration (FPD). FPD measurements were normalized (corrected FPD [cFPD]) to the beating rate of the contracting areas with the Bazzet correction formula: cFPD_FPD/√(RR interval) as described previously [35].

### Statistical Analysis

Comparisons at each time point were conducted using analysis of variance (ANOVA) followed by post-hoc test, and all data are presented as mean values ± S.E.M. Differences were considered statistically significant at p≤0.05.

## Results

### Generation and Characterization of human iPSCs

**Figure 1:**
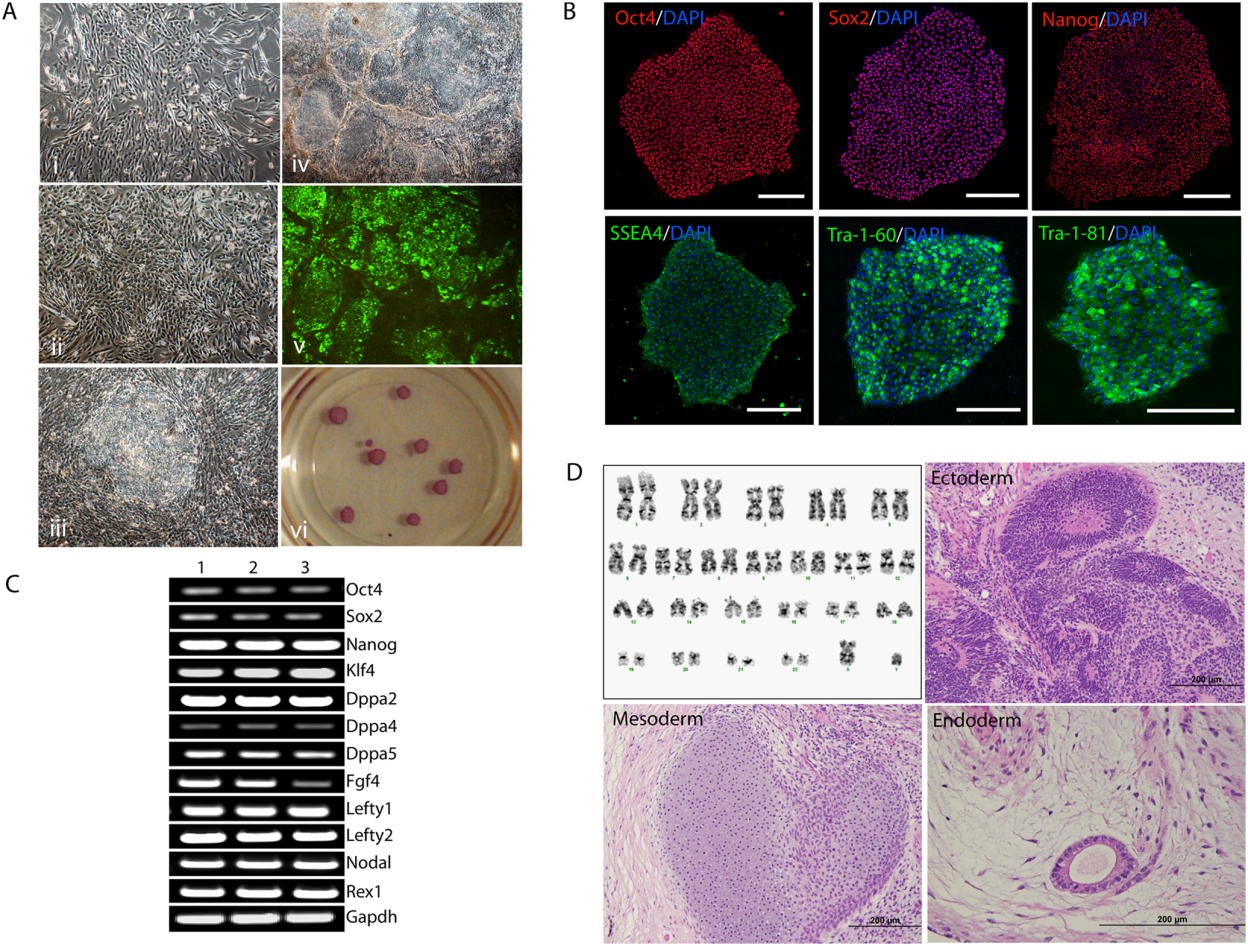
Characterization of transgene free iPSC. A, Micrographs showing morphological changes during mRNA reprograming on day 6 (i), 8 (ii), 15 (iii) and 20 (iv) with live Tra-1-60 (v) staining (day 20). vi, image shows hiPSCs positively stained with alkaline phosphatase. B, Immunostaining of the undifferentiated hiPSC colonies with Oct-4, Sox2, Nanog, SSEA-4, Tra-1-60 and Tra-1-81 antibodies followed by counterstaining with DAPI. Scale bar –200 µm. C, Semi-quantitative gene expression levels of pluripotency associated genes in two hiPSC clones (lane 2 and 3) in comparison with hESC line (lane 1, positive control) with GAPDH as internal loading control. D, A typical normal karyogram of hiPSC clone 1 and Hematoxylin and eosin (H&E) staining of teratoma sections of clone 1 showing the presence of ectoderm (neural rosettes), mesoderm (cartilage) and endoderm (secretory tubule). Scale bar –200 µm.

We generated transgene-free human hiPSC clones utilizing mRNA reprograming techniques. First visual changes were clearly observed post transfection day 6 (Figure 1Ai) and these changes became more prominent post day 8 (Figure 1Aii), where small cellular aggregations started to appear. Human hiPS colony-like structures were clearly identifiable by day 15 of consecutive transfections (Figure 1Aiii). Live Tra-1-60 staining was performed on day 20, to select prospective hiPSC clones and majority of the colonies stained positively for Tra-1-60 antigen (Figure 1Aiv–v). A total of 12 clones were randomly picked and stained positive for alkaline phosphatase (Figure 1Avi), confirming that all picked clones were hiPSCs. Visual morphology of these hiPSCs was similar to hESC, with compact colonies, high nucleus-to-cytoplasm ratios and prominent nucleoli on feeders as well as feeder-free culture. Out of the 12 clones, 2 clones were propagated for further characterizations. Our immunostaining results showed that both clones stained positively for pluripotency transcriptional factors (Oct-4, Sox2 and Nanog) and surface antigens (SSEA4, Tra-1-60 and Tra-1-81) (Figure 1B, Figure S1 in File S1). Moreover, semi-quantitative gene expression for a panel of 12 pluripotency associated markers demonstrated that these hiPSC clones expressed similar levels of all markers when compared to standard hESC cells (Figure 1C). Both clones maintained a normal karyotype (Figure 1D and supplementary figure 1) and transplantation of undifferentiated cells in SCID mice generated teratoma at 8 weeks. H and E staining of teratoma sections demonstrated the presence of neuroepithelial rosettes (ectoderm), cartilage/adipose (mesoderm) and secretory tubules (endoderm), representing the three germinal layers (Figure 1D and figure S1 in File S1).

In order to validate the protocol of mRNA reprograming, skin fibroblasts from a 57 year old, male cardiac patient with no specific genetic pre-disposition were subsequently reprogrammed. On day 25 of reprogramming, prospective iPSC colonies emerged and 3 clones were picked manually from these prospective colonies on day 27 (figure S2 in File S1). We characterized one clone by immunostaining for the expression of pluripotency markers. Results indicated that the colonies expressed the conventional pluripotency markers (Oct-4, SSEA4, Tra-1-60 and Tra-1-81) (figure S2 in File S1).

### In vitro Cardiac differentiation

To induce cardiac differentiation, 3-dimensional differentiating cell aggregates (EBs- embryoid bodies) were generated from hiPSCs for 8 days. EBs were plated and rhythmically contracting areas appeared by day 11–14 post-differentiation. The number of beating clusters increased with time and differentiation efficiency of our clones based on beating frequency ranged 55–75% by day 21 of differentiation and these contracting areas continued to beat for several weeks (50–60 days) post differentiation. There was not much difference in the contraction efficiencies of clones generated from the two fibroblasts. Furthermore, no significant difference was noted between the two clones generated from BJ fibroblasts. Thus, we selected one clone each from both the commercial and patient fibroblast for downstream experiments.

**Figure 2:**
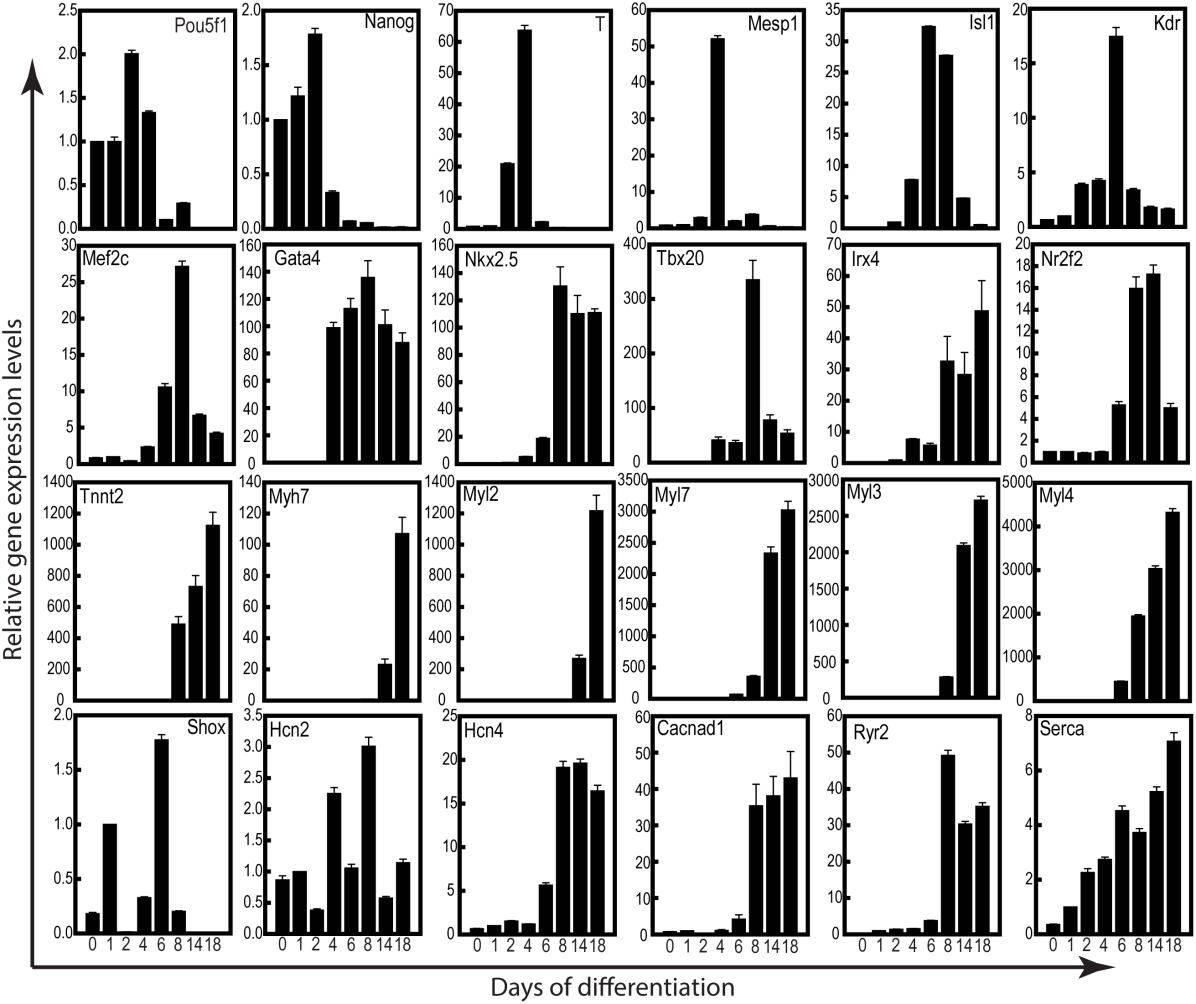
Temporal quantitative gene expression pattern during cardiomyogenesis. Graphs show real-time RT-PCR data showing various hallmark markers for cardiomyocyte differentiation (day 0, 1, 2, 4, 6, 8, 14 and 18). The mean Ct values of duplicate measurements were calculated and subsequently normalized against housekeeping gene (GAPDH) for the same sample. After normalization, the means of triplicate samples from three independent experiments were plotted relative to the day 0 for undifferentiated markers and with day 1 for differentiated markers. Data represented are mean ± SEM (n = 3).

We performed real-time kinetic (day 0, 1, 2, 4, 6, 8, 14 and 18) gene expression studies on a panel of 24 makers to study the developmental ontogeny of these myocytes from mRNA reprogramed hiPSCs (Figure 2). Our gene expression studies indicated that initiation of differentiation was accompanied with a significant decrease in pluripotency associated markers (Oct4, Sox2 and Nanog) by day 6, but there was significant increase in cardiac mesodermal commitment with increased expression of T and Mesp1 by day 4. This spiked increase was subsequently followed by a significant increase in various cardiac transcriptional factors like Isl1, Kdr, Mef2C, NKx2.5, Gata4, Tbx3/5/20 by day 6 onwards. While some makers were transiently up regulated like Isl1, Kdr and Mef2C, other, NKx2.5, Gat4 and Tbx-family members were more consistently expression throughout the differentiation (till day 18). A significant increase was also noted in ventricular and atrial specific transcriptional factor, Irx4 and Nr2f2, respectively post day 8 of differentiation. However, by day 18 of differentiation, Nr2f2 levels decreased, but Irx4 levels increased significantly (Figure 2). Concomitant to increased expression of various cardiac transcriptional factors, significant up regulation were also observed for cardiac specific structural and sarcomeric proteins (Tnni2, Mlc2a/v, Myh7, Myl3/4) by day 8 or 14 of differentiation. Furthermore, ion channel proteins, Ca_v_1.3 encoding the α-1D subunits of L-type calcium channel, (Cacnad1), the hyperpolarization-activated cyclic nucleotide-gated potassium channel (Hcn4) responsible for the I_f_ pacemaker current, sarcoplasmic reticulum Ca^2+^ ATPase (Serca2a) and Ryanodine receptor 2 (Ryr2) were significantly up-regulated by day 14 of differentiation (Figure 2). However, Hcn2 and Shox, both markers of nodal/pacemaker cells did not show significant change in gene expression profile (Figure 2). Interestingly, a similar trend was also observed in the patient fibroblast derived hiPSC-CMs (Supplementary figure 3). Down regulation of pluripotency markers resulted in commitment towards cardiac mesodermal lineage and subsequent increase in cardiac transcriptional factors followed by mature cardiac markers. Although there were subtle changes in relative gene expression between the two cell lines, the general trend towards cardiomyocyte specification remained constant (figure S3 in File S1). Since the trends between both lines were similar, we then focused on BJ-hiPSC clone for subsequent studies.

**Figure 3:**
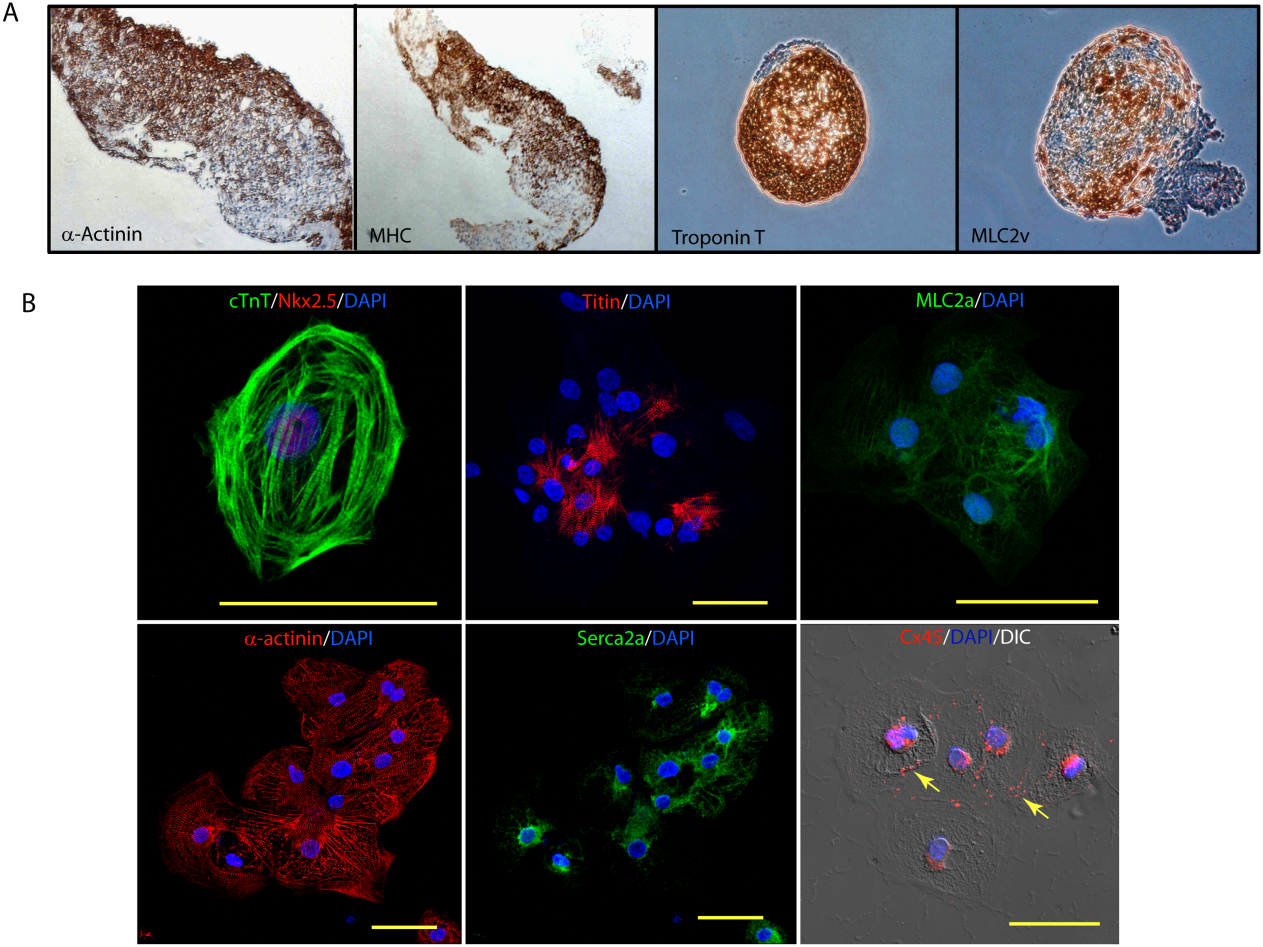
Structural and molecular characterization of iPSC-CM. A, Immunohistochemisty staining of α-actinin, MHC, troponin T and MLC2v on day 18 EB. B, Immunofluorescence images of dissociated EBs for transcriptional factor, Nkx2.5, structural proteins, cardiac troponin-T (cTnT), titin, myosin light chain 2a (MLC2a), sarcomeric α-actinin, ion channel, sarco(endo)plasmic reticulum Ca^2+^-ATPase (SERCA2a) and gap junction connexin 45 (Cx45). Nuclei were counterstained with DAPI in all images. Scale bar: 50 µm.

We next checked for the spatial organization of BJ-hiPSC derived cardiomyocytes by immunostaining. Immunohistochemistry of EB section on day 18 for mature cardiomyocytes markers (α-actinin, myosin heavy chain (MHC), troponin T and MLC2v) showed large portion to be positive for these markers (Figure 3A). Furthermore, dissociated clusters of hiPSC-CMs stained positive for cardiac structural proteins, troponin I, titin, myosin light chain 2a (Mlc2a) and cardiac α-actinin, (Figure 3B). To get a better understanding of differentiation efficiency, we performed a manual count on the number of cells positive for cardiac α-actinin (162/493 DAPI positive cells; 32.8%) and troponin I (136/427 DAPI positive cells; 31.8%). Positively stained cardiomyocytes demonstrated an immature striated pattern indicating towards early stages of myocyte development. However, Z-bands and A-bands were clearly visible in the cardiomyocytes (Figure 3B). Our hiPSC-CMs also stained positively for sarco(endo)plasmic reticulum Ca^2+^-ATPase (SERCA2) and connexin 45 (marked by arrows; Figure 3B) suggesting their ability to regulate calcium [36] and form cell-cell interactions [37].

### Functional characterization of hiPSC-CMs

**Figure 4:**
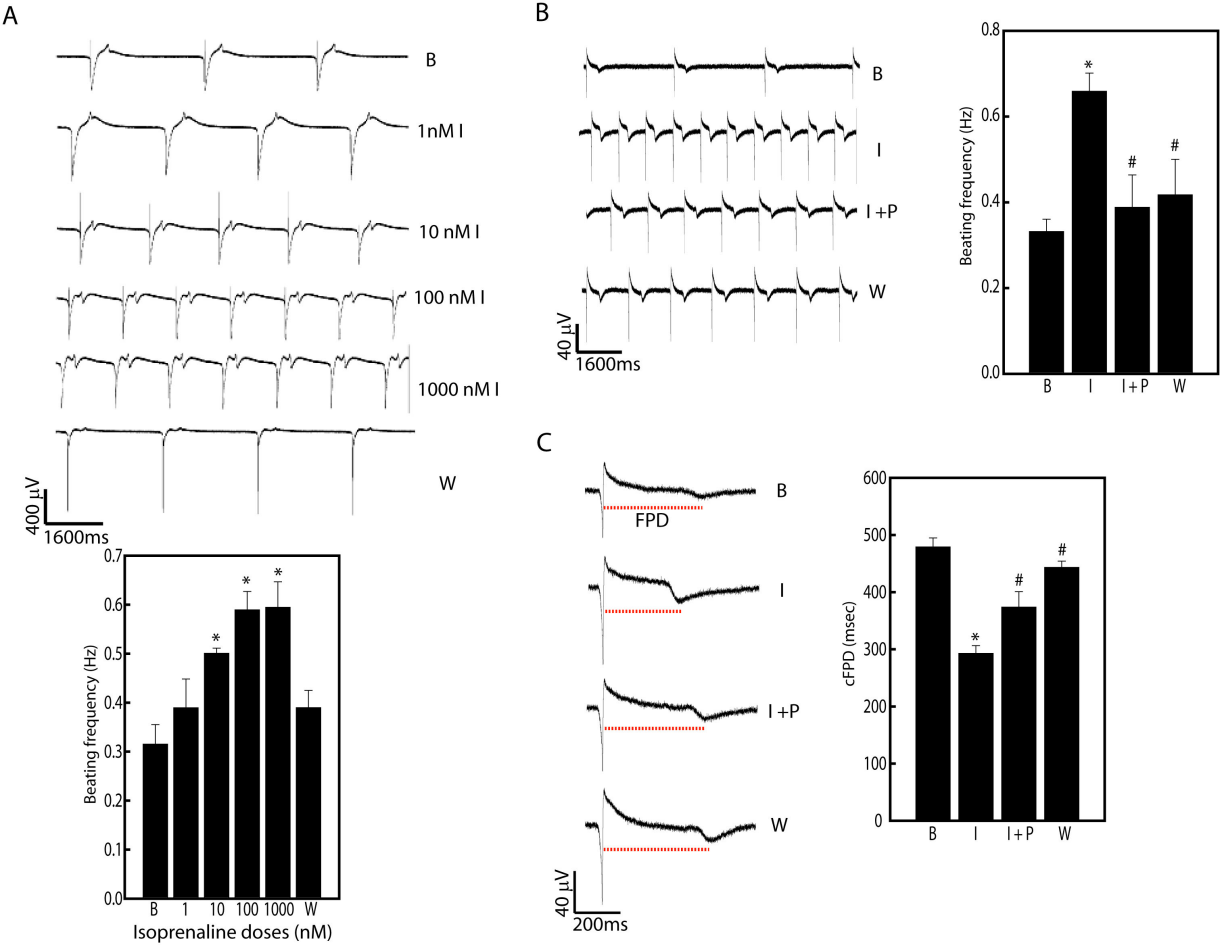
Effects to adrenergic stimulation on cardiomyocytes. A, Multielectrode array (MEA) tracing of dose dependent effects isoproterenol (1–1000 nM) on beating frequency (Hz) on hiPSC derived cardiomyocytes. Note a significant increase in beat rates with increasing dose. *p<0.05 vs control (baseline). Data represented as mean ± SEM of three independent experiments. B, β-adrenergic (propanolol) inhibition of beating frequency under adrenergic stimulation (isoproterenol). Note propranolol (2000 nM) significantly reduced beating frequency post isoproterenol (100 nM) stimulation. C, Alteration in corrected field potential durations (cFPD) in propranolol inhibitor under isoproterenol stimulation. Note isoproterenol reduced cFPDs that reverse with propranolol. *p<0.05 vs control (baseline) and ^#^p<0.05 vs Isoproterenol group. Data represented as mean ± SEM of three independent experiments. The dotted line (red) shows field potential durations in each trace. Abbreviations: I- Isoproterenol; P- Propranolol; B- Baseline; W- Washout.

While these myocytes exhibited hallmark molecular and cellular characteristics, did these hiPSC-CMs display functional electrophysiological properties? We evaluated the ability of these myocytes to respond to pharmacological stimuli. Human iPSC-CMs demonstrated a dose dependent increase in spontaneous contraction frequencies following treatment with β-adrenergic agonist, isoproterenol (Figure 4A). This increase in beating frequency by isoproterenol (100 nM) could be significantly reduced by β-blocker, propranolol (2000 nM) (Isoproterenol vs Isoproterenol + Propranolol (Hz): 0.66±0.04 vs 0.39±0.07, n = 3, p<0.05; Figure 4B) as well as muscarinic inhibitor, carbamycholine (2 µM) (Isoproterenol vs Isoproterenol + Carbamylcholine (Hz): 1.36±0.14 vs 1.03±0.13, n = 3, p<0.05; figure 4 in File S1). Moreover, these changes in beating frequency also affected the field potential durations. While isoproterenol decreased the corrected field potential duration (cFPD) by about 40%, treatment with propranolol reversed the decreased cFPD by 35% (Figure 4C). These results demonstrated that our cardiomyocytes showed apt responses to chronotropic stimulation.

**Figure 5:**
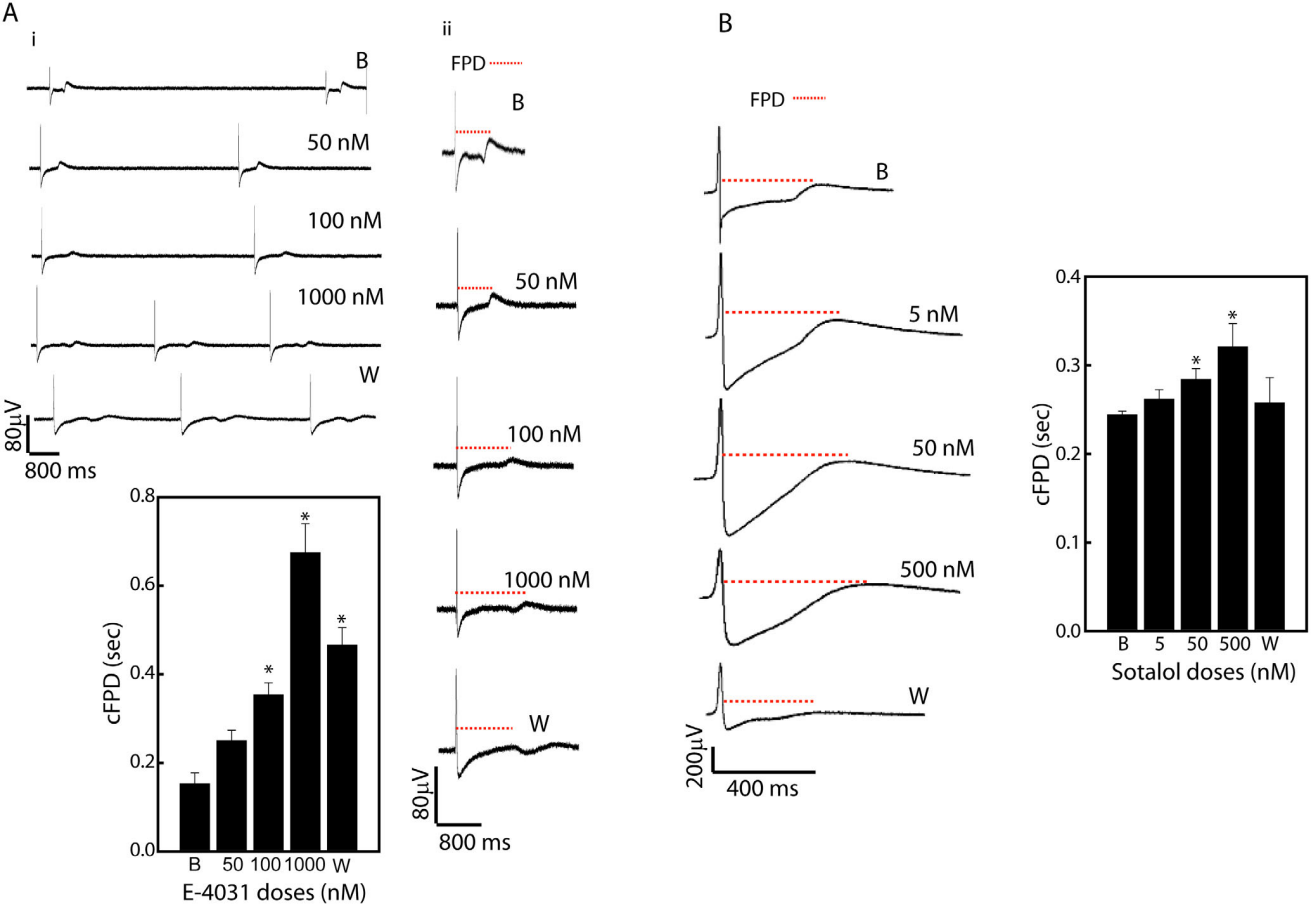
Effect on potassium channel functioning in hiPSC-CMs. Ai, Multielectrode array (MEA) tracing of dose dependent effects E-4031 (50–1000 nM) on changes in cFPD (msec) in hiPSC derived cardiomyocytes over time. ii, enlarged view of a single waveform following E-4031 treatment. *p<0.05 vs control (baseline). Data represented as mean ± SEM of three independent experiments. B, Field potential waveforms following dose dependent treatment with Sotalol (5–500 nM). Note the significant increase in the FP duration following treatment with E-4031 and Sotalol. *p<0.05 vs control (baseline). Data represented as mean ± SEM of three independent experiments. The dotted line (red) shows field potential durations in each trace. Abbreviations: B- Baseline; W- Washout, FPD- field potential duration.

We next evaluated the potassium channels of these myocytes by treatment with 2 class III anti-arrhythmics, E-4031 and Sotalol. Dose dependent treatment with E-4031 caused significant increase in FPD intervals, with its maximal effect at 1000 nM concentration (Baseline vs 1000 nM E-4031(msec): 0.15±0.02 vs 0.67±0.06, n = 3, p<0.05). Similar increase in FPDs was also observed post Sotalol treatment; however the increase was not comparable to E-4031 (Figure 5B). Moreover, further increase in the Sotalol concentration resulted in ventricular trachycardia-like waveforms with significant increase in beating frequencies, ultimately resulting is cardiac arrest (data not shown).

**Figure 6:**
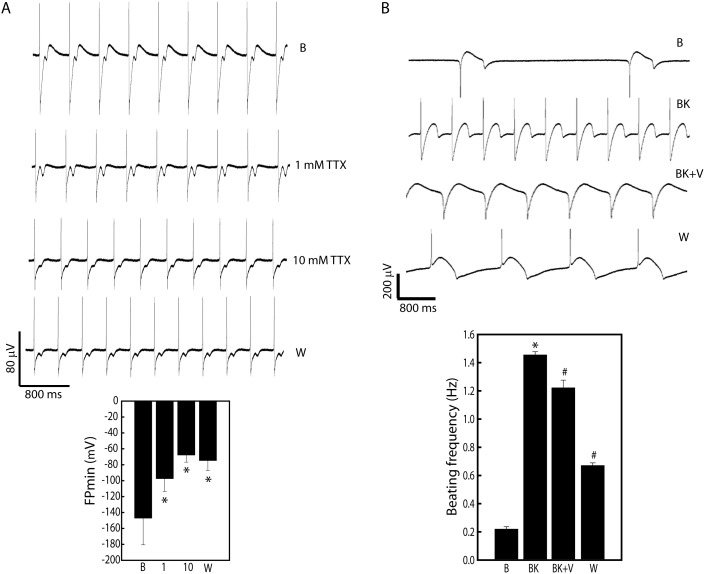
Effects of sodium and calcium channel activity alterations in hiPSC-CMs. A, Extracellular FP recordings following dose dependent treatment of tetradotoxin (TTX, 1 and 10 µM), a sodium channel blocker reduces FP_min_. Note the significant decrease in FP_min_ with TTX. *p<0.05 vs control (baseline). Data represented as mean ± SEM of three independent experiments. B, Activation and inhibition changes in beating frequency by verapamil under Bay K8644 activation. *p<0.05 vs control and ^#^p<0.05 vs Bay group. Data represented as mean ± SEM of three independent experiments. Abbreviations- BK- Bay K8644; V- Verapamil; B- Baseline; W- Washout.

Lastly, we explored the functioning of the sodium and calcium channels in these myocytes. Dose dependent treatment of Tetrodotoxin (TTX), a potent Na^+^ channel blocker, significantly shortened the FP_min_ by 50–55% at a dose of 10 µM (Baseline vs 10 µM TTX (µV): −147.1±33.4 vs −69.4±8.86, n = 3, p<0.05), indicating decreased sodium flux (Figure 6A). On the other hand, nifedipine, a dihydropyridine calcium channel blocker that specifically blocks L-type calcium, significantly reduced the beating frequencies in a dose dependent manner (Baseline vs 10 nM Nif vs 100 nM Nif: 1.77±0.02 Hz vs 1.31±0.07 Hz vs 0.97±0.04 Hz, n = 3, p<0.05). Interestingly, a 1 µM dose of nifedipine resulted caused cessation of spontaneous contractions, however, withdrawal of the drug re-initiated contractions. Activation-inhibition studies demonstrated that 1 µM Bay K8644 (a calcium channel agonist) significantly increased the beating frequency, which could be reduced with 2 µM verapamil (Figure 6B). Nevertheless, these results validated the presence of functional ion channels on these hiPSC-CMs.

## Discussion

The ability to reprogram terminally differentiated somatic cells to pluripotency state holds great promise for clinical applications, drug discovery and developmental biology. Reprogramming technology coupled with generation of patients-specific iPSCs could potentially offer generation of clinically useful cell type for autologous cell therapy. Half a decade ago, Yamanaka and colleagues demonstrated that induction of iPSC is possible by ectopic expression of pluripotency factors using retroviral vectors [1]. While viral delivery systems exhibited high reprogramming efficiencies, integration of the viral genome with the host presents a daunting hindrance for their therapeutic applications. Over the last few years, many studies have shown that pluripotency in somatic cells can be induced through multiple methods that prevent genetic integration of the vectors in the host, overcoming the technical challenges of viral delivery systems in regenerative medicine. While, the efficiency of non-viral methods, such as episomal, plasmid or protein based methods is relatively low, mRNA based methods have shown to have high efficiency and also obviate the need to stringent biological containment, making reprogramming more accessible for clinical setups [25].

While induction of pluripotency is an important aspect for generating patient specific iPSC, the ability of these iPSC to differentiate into high quality of desired cell types like cardiomyocytes is a key feature for regenerative applications. Many reports have indicated that induction of pluripotency is feasible using mRNA methods [25]–[28], however, none of the studies have performed systematic evaluation of their differentiation potential towards cardiomyocyte lineage. In this study, we reprogrammed human fibroblasts using mRNA method (Figure 1) and differentiated them towards cardiac lineage. Our results show that somatic cells could be reprogrammed by daily transfection of mRNA cocktail for 15 days. Although, transfection on a daily basis was labor intensive as compared to other methods like episomal vectors, the clones generated through mRNA appeared earlier (20 days vs 35 days for episomal) and were more abundant as compared to our previous experience with episomal based methods [21], [31]. Human iPSC clones generated demonstrate apt morphological (compact colonies and high nucleus to cytoplasmic ratio) and expression of pluripotency profiles similar to hESC (Figure 1). Importantly, cardiomyogenesis from hiPSCs mimicked human cardiac developmental pathway and resulted in functional cardiomyocytes that responded pertinently to pharmacological compounds.

We evaluated a panel of 24 markers to study the cardiac developmental cascade from hiPSCs (Figure 2). Down-regulation of pluripotency markers, Oct-4 and Nanog, were indicative of differentiation in these pluripotent stem cells [33]. This down-regulation was simultaneously associated formation of primitive streak, as indicated by T up-regulation by day 2 to 4. Furthermore, concomitantly Mesp1, a marker cardiac mesodermal commitment too was up-regulated. Following induction of cardiac mesoderm by day 4, a cascade of cardiac transcriptional factors associated with development like Mef2C, Isl1, GATA4, NKx2.5, Irx4 and Nrf2f indicating commitment towards cardiac fate. Lastly, increased transcripts of cardiac specific structural and ion channel genes, confirmed terminal differentiation. These observations are in concordance with previously published literature based on EB differentiation [35], [38]. These results together suggest that in vitro EB differentiation recapitulates the development pathways [39], that could have important implications in future studies for a better understanding of the signaling pathways responsible for cardiac differentiation. Although, we observed high levels of cardiac related transcripts, however, only 20–35% efficiency in contractions was observed. These observations suggest that the standard SB203580 differentiation protocol utilized may need additional optimizing in order to improve cardiomyocyte yield. In the recent year number of protocols for cardiac differentiation have been developed that utilize 3D differentiation regimes using a combination of small molecules and/or growth factors that help in attaining higher cardiac differentiation efficiencies [38], [40]–[42]. However, culture conditions utilized for maintaining undifferentiated nature of hPSC may also play a vital role in their differentiation abilities [43]. Our group has also demonstrated that timely regulation of Wnt axis could efficiently increase cardiac differentiation efficiencies in a scalable manner using EB based methods [44]. However, most of the myocytes within the EBs areas stained positively for sarcomeric proteins (Figure 3). mRNA iPSC derived cardiomyocytes exhibited Z- and A-bands indicating emerging maturity, however, these sarcomeric proteins were not well organized, and resembled phenotypically to 16-week old fetal hearts [45]. Apart from structural immaturity, number of studies have discussed about electrical immaturity of hiPSC derived myocytes [42], [46]. Human iPSC-CMs demonstrate reduced inward rectifier K current, show presence of prominent pacemaker currents that results in spontaneous depolarizations, which are absent in ventricular myocytes. While intracellular Ca^2+^ stores could be released by hiPSC-CMs with caffeine, most of the Ca^2+^ transients are sensitive to inositol triphosphate [47], not present in adult myocytes [48]. However, we and others have shown that hiPSC-CMs could attain structural maturity on prolonged culturing [32], [49] and may show better electrophysiology [49]. Remarkably, despite immature sarcomeric structures, these cardiomyocytes expressed functionally active ion channels that could be altered by pharmacological agents. Our results using MEA mapping technique show that hiPSC-CMs respond appropriately to compounds known to affect chronotropy, repolarization and QT intervals (Figure 4–6). These results suggest that these cardiomyocytes could be utilized as appropriate human model with high biological relevance in drug safety testing. Moreover, the active presence of these functional ion channels may also help in integrating with the myocardium during autologous therapeutic applications. Although, in this current study we have not addressed the ability of these cardiomyocytes in regenerative medicine, it is likely that these cells that bear no foreign signature will have no immunological responses [50]. Nevertheless, large-scale production, purity, in vivo maturation, electrical activity and arrhythmogenicity are still pressing issues that need to be addressed before mRNA generated hiPSC derived cardiomyocytes could be utilized in the clinics.

In conclusion, we for the first time report a systemic molecular and pharmacological evaluation of mRNA generated iPSC and their differentiation to cardiomyocytes. Our study highlights that differentiation of these hiPSCs towards cardiomyocytes recapitulate cardiac developmental process with well-developed electrical activities. These zero foot print cardiomyocytes may be highly valuable in designing therapeutic regimes and as an in vitro tool for drug discovery.

## Supporting Information

File S1 **This file includes Figures S1 to S3.**
(DOCX)
