# Correction: A Systemic Evaluation of Cardiac Differentiation from mRNA Reprogrammed Human Induced Pluripotent Stem Cells

**DOI:** 10.1371/journal.pone.0114771

**Published:** 2014-11-25

**Authors:** The PLOS ONE Staff

There is an error in affiliation 2 for author William Sun. Affiliation 2 should be: Institute of Bioengineering and Nanotechnology, A*STAR, Singapore, Singapore.

One of the affiliations for the eighth author is not indicated. William Sun is affiliated with #2 and with the Department of Physiology, Yong Loo Lin School of Medicine, National University of Singapore, Singapore.
