# Supplementary material for: A Systemic Evaluation of Cardiac Differentiation from mRNA Reprogrammed Human Induced Pluripotent Stem Cells
Source: PLoS One. 2014 Jul 28;9(7):e103485. doi: 10.1371/journal.pone.0103485 (PMC4113436; doi:10.1371/journal.pone.0103485)
Supplement: File S1 — This file includes Figures S1 to S3. (DOCX) [file pone.0103485.s001.docx]

**Supplementary table 1:** List of primers used in the study

| **Gene name** | **Forward primer** | **Reverse primer** |
| --- | --- | --- |
| *Pou5f1* | AGTTTGTGCCAGGGTTTTTG | ACTTCACCTTCCCTCCAACC |
| *Nanog* | CTCCATGAACATGCAACCTG | GAGGAAGGATTCAGCCAGTG |
| *Sox2* | AAAAATCCCATCACCCACAG | GCGGTTTTTGCGTGAGTGT |
| *T* | GCGCGAGAACAGCACTACTA | GACCAAGACTGTCCCCGCTC |
| *Mesp1* | CGAGTCCTGGATGCTCTCTG | CCATGAGTCTGGGGACGAGA |
| *Isl1* | AAGGACAAGAAGAGAAGCAT | CATGGGAGTTCCTGTCATCC |
| *Kdr* | GGCGGCACGAAATATCCTCT | GGAGGCGAGCATCTCCTTTT |
| *Mef2c* | TGGAGAAGCACTTCAACGCT | TCCTGCATTCGTTCCTGATGA |
| *Gata4* | CGACACCCCAATCTCGATATGTT | ACAGATAGTGACCCGTCCCA |
| *Nkx2.5* | CAAGTGTGCGTCTGCCTTTC | CGCGCACAGCTCTTTCTTTT |
| *Tbx20* | AACCCCAAATCGAGGGTCAG | AAGAGCAGTCAGGGACTGTG |
| *Irx4* | GGCTCCCCAGTTCTTGATGG | CTCGTAGACCGGGCAGTAGA |
| *Nr2f2* | CGCACGAAGGATGTGCTTCTA | TCACACACATAGGGAAAGAGTCA |
| *Tnnt2* | TTACATCCAGAAGACAGAGCGG | GTCAATGGCCAGCACCTTCC |
| *Myh7* | GGCAAGACAGTGACCGTGAAG | CGTAGCGATCCTTGAGGTTGTA |
| *Mlc2v/Myl2* | TGGGCGAGTGAACGTGAAAA | AGGGTCCGCTCCCTTAAGTT |
| *Mlc2a/Myl7* | GGAGTTCAAAGAAGCCTTCAGC | TCCTCTGGGACACTCACCTT |
| *Myl3* | GCCAAGACAGGAAGAGCTCAAT | CCTCATAGGTGCCTGTGTCC |
| *Myl4* | TCATTGTTTGACCGGACCCC | TTCATCTCTTCAGGCTTGGGC |
| *Shox* | AACTCCATAAAGGTGTTCTCATAGG | CCTGCTGAAATGGCATCCTT |
| *Hcn2* | CACCTGCTACGCCATGTTCA | CTGGCAGCTTGTGGAAGGA |
| *Hcn4* | GGGAATTCGCAACTGAAGCC | CGGGGTGAGAGGTATCCACA |
| *Cacna1d* | GGGCAATGGGACCTCATAAATAA | TTACCTGGTTGCGAGTGCATTA |
| *Ryr2* | TGCATGAAAGCATCAAACGCA | TCCACCACACAGCCAATCTC |
| *Serca/* *Atp2a2* | CGAACCCTTGCCACTCATCT | CAGGTTCCAGGTAGTTGCGG |
| *Klf4* | TACCAAGAGCTCATGCCACC | CGCGTAATCACAAGTGTGGG |
| *Dppa2* | TTCCATCCCAGGGTGTTGCT | TTGCGTCATCTTTAACTGGCAC |
| *Dppa4* | CACTGACAACCCCAGACCTC | CCAATTTCTGGCCTTTGGAGC |
| *Dppa5* | TCACCGAGGTCGTGGTTTAC | AGTTTGAGCATCCCTCGCTC |
| *Fgf4* | CTCTATGGCTCGCCCTTCTTC | TGAACATGCCGGGGTACTTG |
| *Lefty1* | AGAGCTGGCGATGACTGAAC | AAACTGAGCAAGGGCTCTCC |
| *Lefty2* | CCCCTGAGAGGGTGCTAAGA | GGAAATGACGAGCCAAAGCC |
| *Nodal* | CCCAAGCAGTACAACGCCTA | ACGTTTCAGCAGACTCTGGAT |
| *Rex1* | GTGGACACGTCTGTGCTCTT | GAGCTGTGCCCATCCACATT |
| *Gapdh* | GTGGACCTGACCTGCCGTCT | GGAGGAGTGGGTGTCGCTGT |


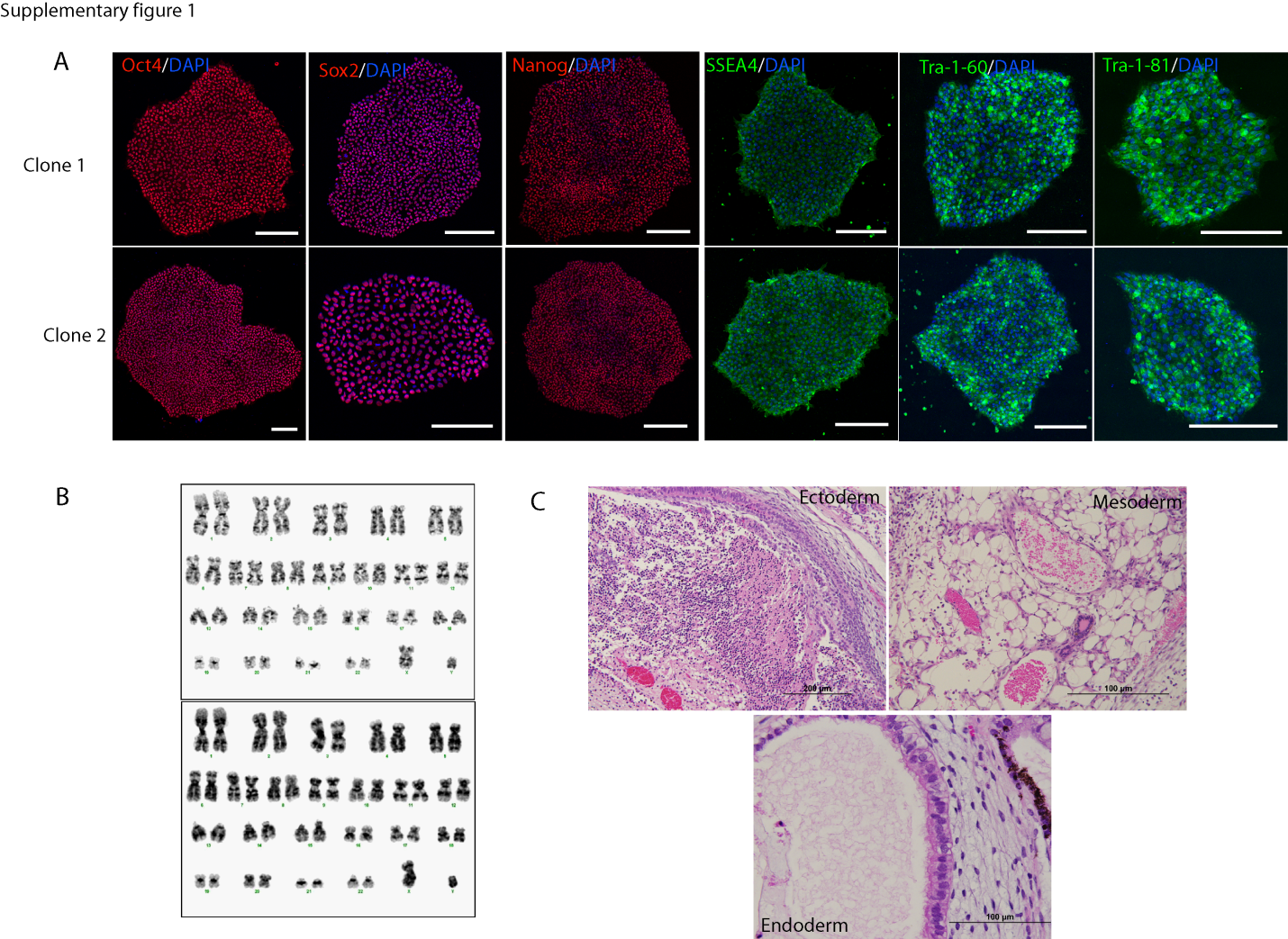


**Supplementary figure 1:** Characterization of mRNA derived hiPSC. A, Immunostaining of the undifferentiated hiPSC colonies with Oct-4, Sox2, Nanog, SSEA-4, Tra-1-60 and Tra-1-81 antibodies followed by counterstaining with DAPI. Scale bar – 200 μm. B, A typical normal karyogram of the two hiPSC clones. C, Hematoxylin and eosin (H&E) staining of teratoma sections of clone 2 showing the presence of ectoderm, mesoderm and endoderm. Scale bar – 200 μm.


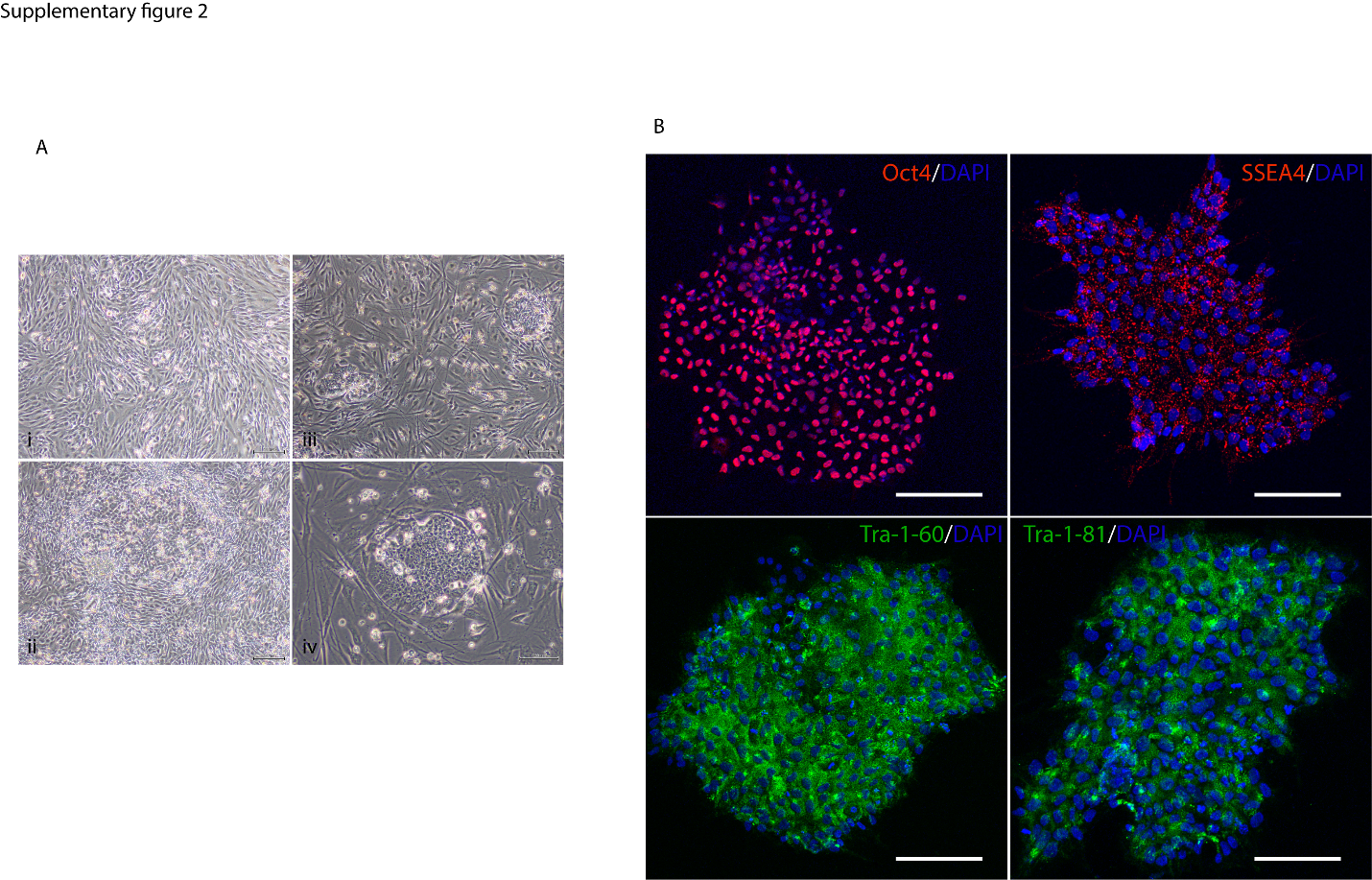


**Supplementary figure 2:** Characterization of normal patient fibroblast derived hiPSC iPSC. A, Micrographs showing morphological changes during mRNA reprograming on day 7 (i), 13 (ii), 18 (iii) and 22 (iv). B, Immunostaining of the undifferentiated hiPSC colonies with Oct-4, SSEA-4, Tra-1-60 and Tra-1-81 antibodies followed by counterstaining with DAPI. Scale bar – 200 μm.


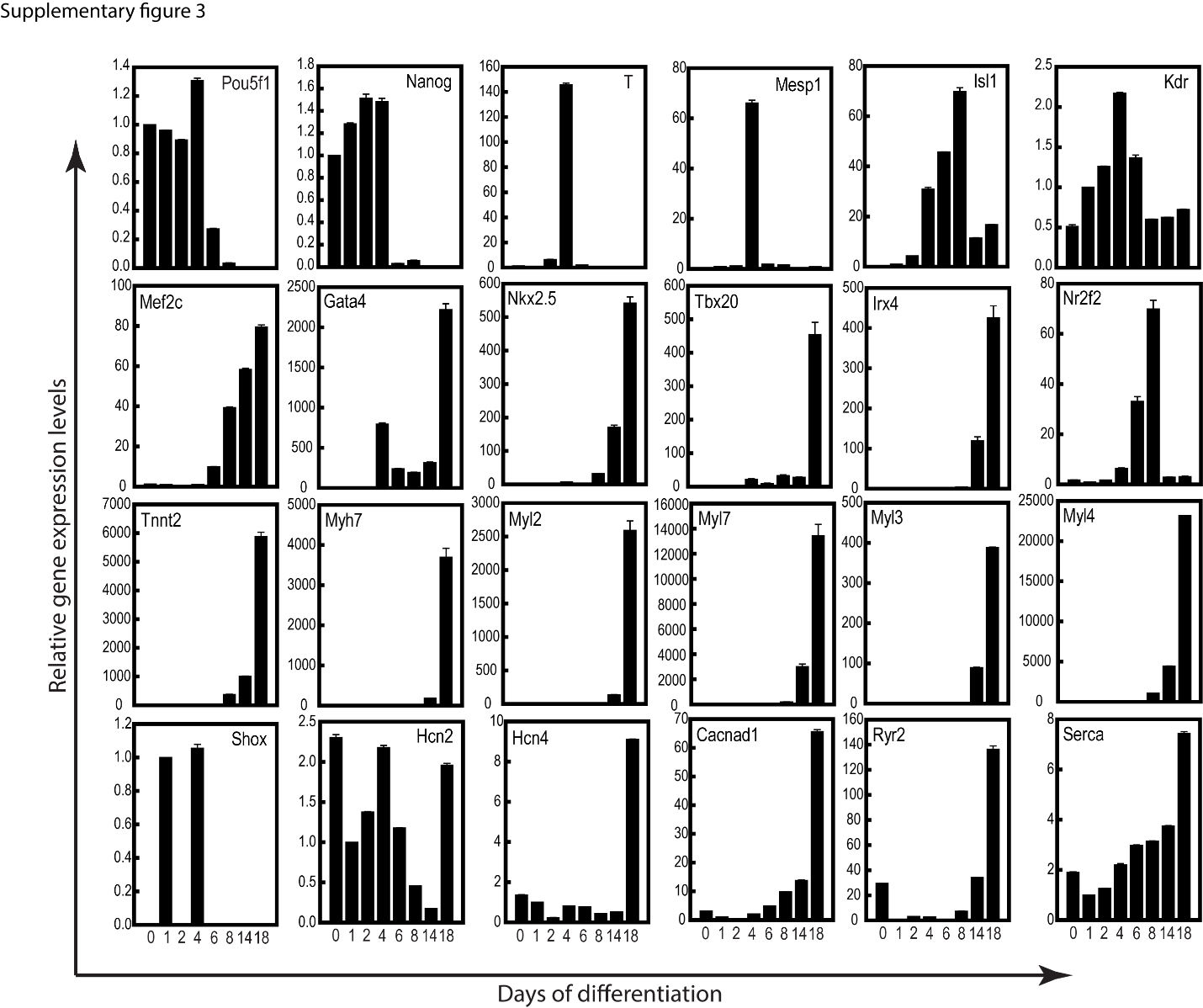


**Supplementary figure 3:** Temporal quantitative gene expression pattern during cardiomyogenesis. Graphs show real-time RT-PCR data showing various hallmark markers for cardiomyocyte differentiation (day 0, 1, 2, 4, 6, 8, 14 and 18). The mean Ct values of duplicate measurements were calculated and subsequently normalized against housekeeping gene (GAPDH) for the same sample. After normalization, the means of triplicate samples from three independent experiments were plotted relative to the day 0 for undifferentiated markers and with day 1 for differentiated markers. Data represented are mean ± SEM (n=3).


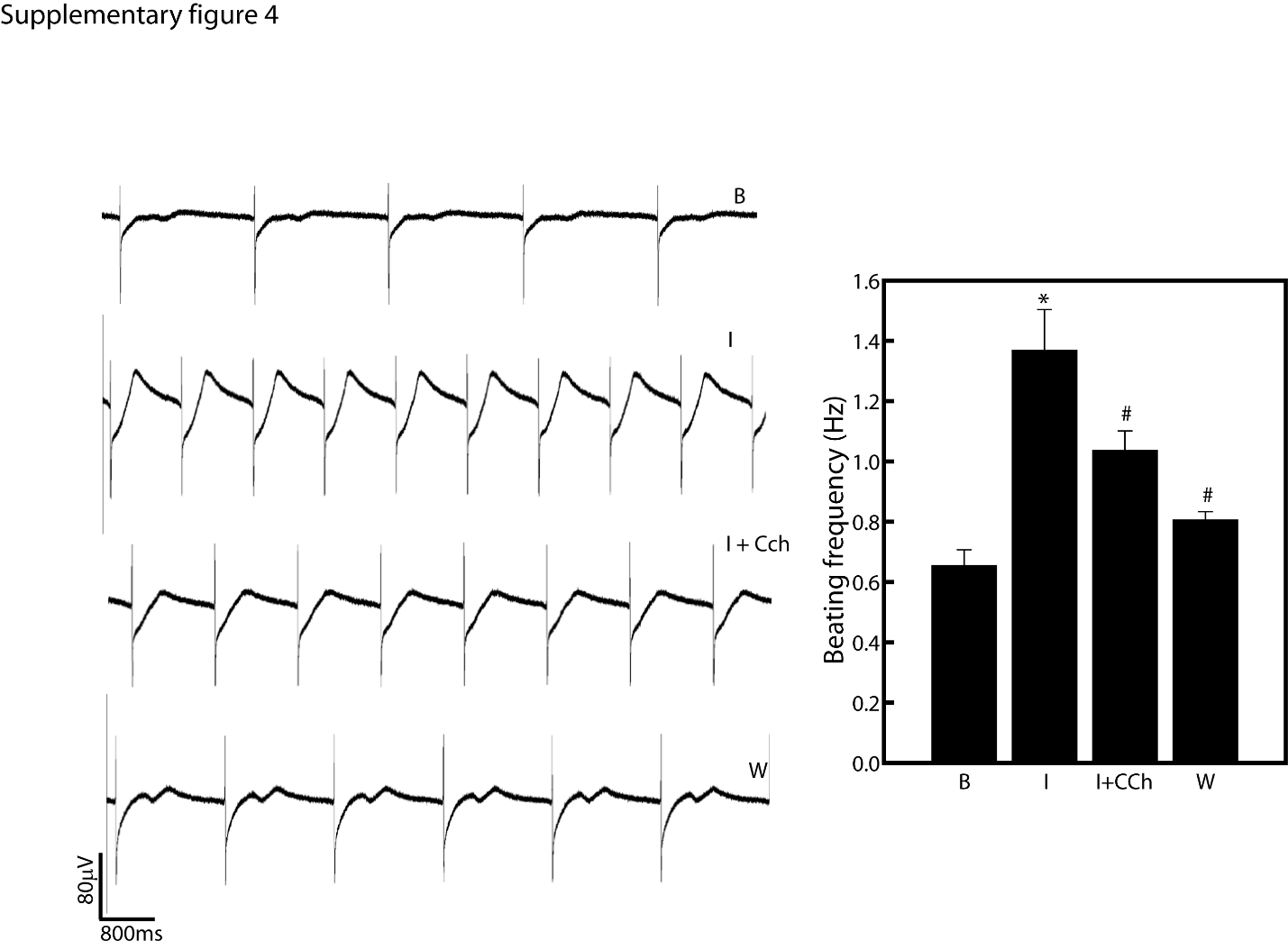


**Supplementary figure 4:** Effects to adrenergic stimulation on cardiomyocytes. A, Muscarine receptor agonist (Carbamylcholine) inhibition of beating frequency under adrenergic stimulation (isoproterenol). Note carbamylcholine (2 µM) significantly reduced beating frequency post isoproterenol (0.1 µM) stimulation. ^*^p<0.05 vs control (baseline) and ^#^p<0.05 vs Isoproterenol group. Data represented as mean ± SEM of three independent experiments. Abbreviations: I- Isoproterenol; CCh- Carbamylcholine; B- Baseline; W- Washout.
